# Hydrogen sulphide ameliorates dopamine‐induced astrocytic inflammation and neurodegeneration in minimal hepatic encephalopathy

**DOI:** 10.1111/jcmm.15728

**Published:** 2020-10-28

**Authors:** Weishan Zhuge, Qichuan Zhuge, Weikan Wang, Xiaoai Lu, Ruimin You, Leping Liu, He Yu, Jian Wang, Xuebao Wang, Yiru Ye, Saidan Ding

**Affiliations:** ^1^ Gastrointestinal Surgery The First Affiliated Hospital of Wenzhou Medical University Wenzhou Zhejiang China; ^2^ Neurosurgery Department The First Affiliated Hospital of Wenzhou Medical University Wenzhou Zhejiang China; ^3^ Department of Microbiology, Immunology, and Genetics University of North Texas Health Science Center Fort Worth TX USA; ^4^ Zhejiang Provincial Key Laboratory of Aging and Neurological Disease Research The First Affiliated Hospital of Wenzhou Medical University Wenzhou Zhejiang China; ^5^ Analytical and Testing Center Wenzhou Medical University Wenzhou Zhejiang China; ^6^ School of Information and Engineering Wenzhou Medical University Wenzhou Zhejiang China

**Keywords:** dopamine, hydrogen sulphide, minimal hepatic encephalopathy, neurodegeneration, TNF‐α

## Abstract

It has been demonstrated that the action of dopamine (DA) could enhance the production of tumour necrosis factor‐α (TNF‐α) by astrocytes and potentiate neuronal apoptosis in minimal hepatic encephalopathy (MHE). Recently, sodium hydrosulfide (NaHS) has been found to have neuroprotective properties. Our study addressed whether NaHS could rescue DA‐challenged inflammation and apoptosis in neurons to ameliorate memory impairment in MHE rats and in the neuron and astrocyte coculture system. We found that NaHS suppressed DA‐induced p65 acetylation, resulting in reduced TNF‐α production in astrocytes both in vitro and in vivo. Furthermore, decreased apoptosis was observed in neurons exposed to conditioned medium from DA + NaHS‐challenged astrocytes, which was similar to the results obtained in the neurons exposed to TNF‐α + NaHS, suggesting a therapeutic effect of NaHS on the suppression of neuronal apoptosis via the reduction of TNF‐α level. DA triggered the inactivation of p70 S6 ribosomal kinase (S6K1) and dephosphorylation of Bad, resulting in the disaggregation of Bclxl and Bak and the release of cytochrome c (Cyt. c), and this process could be reversed by NaHS administration. Our work demonstrated that NaHS attenuated DA‐induced astrocytic TNF‐α release and ameliorated inflammation‐induced neuronal apoptosis in MHE. Further research into this approach may uncover future potential therapeutic strategies for MHE.

## Graphical abstract

XXX.

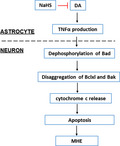

## 1 INTRODUCTION

Endogenous hydrogen sulphide (H_2_S) serves as a gaseous mediator and gasotransmitter signalling molecule^1^ that has multiple regulatory roles in the nervous system of humans and mammals.^2^ A great quantity of testimonies sustain that H_2_S blocks oxidant, inflammatory, and apoptotic activities and effects on neuron,^3^ microglia and astrocytes in vitro.^4^, ^5^ As a physiological product generated in the brain, H_2_S plays a role as an endogenous neuroprotective agent.^6^ Dysregulation of H_2_S homeostasis can contribute to most of the pathological processes of neurodegenerative disorders such as vascular dementia, Alzheimer's disease (AD) and Parkinson's disease (PD).^5^ Meanwhile, it was reported that the intervention of H_2_S could ameliorate the learning and memory impairment and neuroinflammation^7^ by specifically enhancing hippocampal long‐term potentiation.^8^ NaHS, as an exogenous donor of H_2_S, prevents inflammatory response in post‐ischaemic murine small intestine^9^ and inhibits apoptosis by reducing caspase‐3 expression in the mouse brain.^10^

Brain dopamine (DA) has been implicated in cognitive processes such as working memory and cognitive flexibility,^11^ and the DA system is involved in cognitive decline.^12^ Our previous studies have confirmed that the pathogenesis of minimal hepatic encephalopathy (MHE) was caused by the abnormal elevation of intracranial DA.^13^, ^14^, ^15^, ^16^, ^17^, ^18^ It has been demonstrated as a novel regulatory mechanism that DA‐induced astrocytic tumour necrosis factor‐α (TNF‐α) release triggers progressive neurodegeneration, which leads to learning and memory decline in MHE.^19^ Therefore, we suggested that NaHS might have therapeutic effects on DA‐induced cognitive decline by reducing the secretion of astrocytic TNF‐α in MHE rats.

In this study, we mainly focused on investigating whether NaHS had dual effects on the DA‐induced MHE. Firstly, we examined whether NaHS attenuated DA‐mediated TNF‐α release from astrocytes via p65 signalling. Secondly, we assessed whether NaHS reversed TNF‐α induced neuronal apoptosis.

## 2 MATERIALS AND METHODS

### 2.1 MHE models and treatments

Sprague‐Dawley rats (220‐250 g) provided by experimental animals of the Chinese Academy of Sciences in Shanghai were raised according to the instructions of the National Health and Institutional Animal Care and Use Committee of Wenzhou Medical University.

Before the experiment, all animals reached the regular range of the water finding task (WFT) and the Y maze (YM). Animals (n = 30) were injected intraperitoneally (i.p.) with thioacetamide (TAA) (200 mg/kg saline; Sigma‐Aldrich, St. Louis Missouri, USA) twice a week for 8 weeks to induce cirrhosis. Rats treated with TAA exhibited reduced exercise capacity, and rats with lethargy and delayed development of coma were classified as HE.^16^ The MHE status was determined by WFT values above average ±1.96 SD or YM values below average ±1.96 SD after TAA treatment in asymptomatic rats. MHE rats (n = 19) were then anaesthetized with 10% chloral hydrate (0.4 g/kg, i.p.). MHE rats were stimulated i.p. with NaHS (6 mg/ kg) three times at 7‐day intervals. Samples from killed rats were collected after the last dose.

### 2.2 DA‐treated rat models and treatments

Intraventricular injection of DA hydrochloride (10 μg/3 μL in saline) into the left ventricle of anaesthetized rats (anterior and posterior 0.3 mm; lateral 1.0 mm; horizontal, anterior reg 3.0 mm), one time a week for 3 weeks (n = 15). Intraperitoneal (i.p.) injection of NaHS (6 mg/kg) was performed one time a week after the last DA injection for 3 weeks. When the rats were subjected to the YM and WFT tests after the last injection, samples of the killed rats were collected.

### 2.3 Behavioural tests

Arbitrarily placing the rat in the end of the arm in the three‐armed device allowed the rat to freely discover the maze for 8 minutes. The percentage of spontaneous alternation (SA%) was determined by the ratio of arm selection to total selection.^20^ The rat placed in the upper right corner of the WFT instrument was arranged to find and drink the water in the wall within 3 minutes. The elapsed time of entering into the alcove (entry latency, EL), elapsed time of first touching/smelling/licking of the water pipe (contacting latency, CL) and elapsed time of starting drinking from the water pipe (drinking latency, DL) were carried out.^21^

### 2.4 Brain H_2_S detection

The isolated brain tissue was separated in a non‐retractable separation buffer (50 mmol/L Tris‐HCl pH = 8.0, 150 mmol/L NaCl, 1% Nonidet P‐40, 1% Triton X‐100, protease inhibitors) and was homogenized on ice for 20 minutes and then centrifuged at 14 000× g for 15 minutes at 4°C. Protein extract was reacted with reaction mixture (10 μmol/L H_2_S probe 7‐azido 4‐methylcoumarin, 1 mmol/L homocysteine, 50 μmol/L tolaldehyde‐5ʹ‐phosphate, 1 mmol/L L‐cysteine and 50 mmol/L Tris‐HCl) at 37°C for 2 hours, and the fluorescence analysis was performed using a Synergy H1 Hybrid Reader (Bio‐tek Instruments, Winooski, VT, USA) with ex = 365 nm and em = 450 nm. A linear standard curve was acquired by addition of NaHS into reaction mixture.

### 2.5 Determination of DA levels

Serum or lysates of the liver/hippocampus were then collected and analysed for quantitative determination of DA using a commercial DA ELISA assay kit (Cusabio, Wuhan, China). Data analysis was performed by microtiter plate reader (BIO‐TEK Synergy 2). The results presented are from three independent experiments (n = 3).

### 2.6 Culture and treatments of primary hippocampal and cortical astrocytes (PHAs and PCAs)

Primary hippocampal astrocytes (PHAs) or Primary cortical astrocytes (PCAs) were isolated from hippocampus or cerebral cortex tissues of 1‐day‐old SD rat pups by mechanical digestion. 11 mL of 1% serum‐containing DMEM/ F12 medium was used to incubate cells seeded in 75 cm^2^ tissue culture flasks at a density of 15 × 10^6^ cells for 72 hours. The medium was changed every 72 hours. After 7 days of incubation in the primary culture, oligodendrocytes were separated from astrocytes by shaking at 200× g for 18 hours at 37°C. Cells were plated in a six‐well plate coated with poly‐L‐lysine after incubation for 7 days in a new 75 cm^2^ flask. Treatment of astrocytes with NaHS (50, 100 or 300 μmol/L) was performed in a pre‐incubation of DA (final concentration 10 μmol/L) or TNF‐α (final concentration of 1 ng/mL) for 24h. After changing the medium with serum‐free DMEM/ F12 for 6‐8 hours, conditioned glial medium (GCM) was collected. The collected GCM was centrifuged at 1000× g for 4 minutes, and then 10 times concentrated with the Amicon Ultra‐4 filter device (Millibo, Billy Rica, MA, USA).

### 2.7 Culture and treatments Primary hippocampal and cortical neurons (PHNs and PCNs)

PHNs or PCNs were gained from the hippocampus or cerebral cortex of 1‐day‐old SD rat pups. The tissue was mechanically digested in medium containing trypin and DNase, and cells at the density of 2 × 10^6^ cells/well were inoculated in a poly‐L‐lysine‐coated six‐well plate with Neurobasal^®^ medium (1×) contained with 0.5 mmol/L GlutaMAX™‐I, B‐27^®^. After 4 days, cells were changed with media and then treated with NaHS (50, 100 or 300 μmol/L) in pre‐incubation of TNF‐α (final concentration of 1 ng/mL) for 24 hours. Otherwise, PHNs were incubated with GCM from PHAs treated with vehicle (GCM_PHAs‐vehicle_), GCM from PHAs treated with DA (GCM_PHAs‐DA_), GCM from PHAs treated with 50 μmol/L NaHS in pre‐incubation of DA (GCM_PHAs‐DA‐50NaHS_), GCM from PHAs treated with 100 μmol/L NaHS in pre‐incubation of DA (GCM_PHAs‐DA‐100NaHS_), or PHAs treated with 300 μmol/L NaHS in pre‐incubation of DA (GCM_PHAs‐DA‐300NaHS_); and PCNs were incubated with GCM from PCAs treated with vehicle (GCM_PCAs‐vehicle_), GCM from PCAs treated with DA (GCM_PCAs‐DA_), GCM from PCAs treated with 50 μmol/L NaHS in pre‐incubation of DA (GCM_PCAs‐DA‐50NaHS_), GCM from PCAs treated with 100 μmol/L NaHS in pre‐incubation of DA (GCM_PCAs‐DA‐100NaHS_), or GCM from PCAs treated with 300 μmol/L NaHS in pre‐incubation of DA (GCM_PCAs‐DA‐300NaHS_).

### 2.8 Thiazolyl Blue Tetrazolium Bromide (MTT) assay

A stock Thiazolyl Blue Tetrazolium Bromide (MTT_ solution (1 mg/mL in distilled water) was prepared immediately prior to use and filtered through a 0.22 μm Millipore® filter. The culture medium of each cell culture was replaced by 1.5 mL of complete DMEM, to which 0.1 mL of the MTT stock solution was added (final MTT concentration: 62.5 μg/mL). Cells were incubated for 1‐4 hours at 37°C, after which the medium was removed and the culture washed with PBS. For the viability assay, the formazan product was dissolved in 1.5 mL dimethylsulphoxide and the absorbance measured at 540 nm with a plate reader (SpectraFluor; Tecan, Männedorf, Switzerland). Cell survival was expressed as the percentage of formazan absorbance. Results were the mean values and SD from at least three different experiments in triplicate.

### 2.9 ELISA assay

Tumour necrosis factor‐α levels in 96‐well plates were detected using a high‐sensitivity sandwich ELISA kit using a Thermo Fisher Multiskan MC plate reader for spectrophotometry in accordance with the instructions from Promega and SydLabs.

### 2.10 Semi‐quantitative PCR

Total RNA was extracted using TRIzol reagent (Invitrogen, Carlsbad California, USA). Total reverse transcriptase (Quiagen, Hilden, Germany) was used to synthesize cDNA. The cDNA template was subjected to using Taq DNA polymerase (Sigma‐Aldrich) for PCR amplification under conditions of denaturing at 94°C for 30 seconds, annealing at 60°C for 30 seconds and extending at 72°C for 90 seconds. The data of Real‐time PCR were standardized to GAPDH mRNA. PCR primers are listed in Table 1.

**TABLE 1:** Sequences of used primers

Gene name	Sequence	
TNF‐α	5′‐TGAGCACAGAAAGCATGATC‐3′	Forward
	5′‐CATCTGCTGGTAC CACCAGTT‐3′	Reverse
Caspase 3	5′‐GACGTGGATGCAGCAAACCTCA‐3′	Forward
	5′‐TTCACCATGGCTTAGAAGCACG‐3′	Reverse
Caspase 8	5′‐CTGCTGGGGATGGCCACTGTG‐3′	Forward
	5′‐TCGCCTCGAGGACATCGCTCTC‐3′	Reverse
Caspase 9	5′‐GCGTCCATCTGGTCATCTATTCC‐3′	Forward
	5′‐ACACATTGGGGGTAGGAACA‐3′	Reverse
MAP2	5′‐CTGGACATCAGCCTCACTCA‐3′	Forward
	5'‐GCCTTCCTCCTCCTCTCTGT‐3′	Reverse
GFAP	5′‐GAGTTACCAGGAGGCACTCG‐3′	Forward
	5′‐ATGGTGATGCGGTTTTCTTC‐3′	Reverse
IBA1	5′‐CCATGAAGCCTGAGGAAATTTCA‐3′	Forward
	5′‐TTATATCCACCTCCAATTAGGGCA‐3′	Reverse
CD31	5′‐AGTGAGGTTCTGAGGGTGAAGG‐3′	Forward
	5′‐TCACTCCGATGATAACCACTGC‐3′	Reverse
O4	5′‐ACTATGGTTTGGCTATACTCCT‐3′	Forward
	5′‐ATTCATATCCTGCGTGGC‐3′	Reverse
S6K1	5′‐AAATGCTGCTTCTCGTCT‐3′	Forward
	5′‐GTTCTTCCCAGTTAATATGTCT‐3′	Reverse
GAPDH	5′‐ACCCAGAAGACTGTGGATGG‐3′	Forward
	5′‐ACACATTGGGGGTAGGAACA‐3′	Reverse

Abbreviation: TNF‐α, tumour necrosis factor‐α.

### 2.11 Immunoblotting

Homogenized tissues or cells were lysed by using RIPA lysis buffer (50 mmol/L Tris‐HCl (pH 7.4), 150 mmol/L NaCl, 1% Triton‐X100, 1 mmol/L Phenylmethylsulfonyl fluoride (PMSF), 2 μg/mL aprotinin, 2 μg/mL leucine, 1.5 mmol/L Ethylene Diamine Tetraacetic Acid (EDTA)). Protein was separated using SDS‐PAGE and then electroblotted onto Polyvinylidene Fluoride (PVDF) membranes (Millipore, Bedford, MA, USA). After blocking with 5% (w/v) skimmed milk powder, the protein was detected overnight at 4°C with primary antibodies (TNF‐α, Caspase3, Caspase9, cytochrome c [Cyt.c], phospho‐Bad [pBad]). The membrane after several washes was incubated with a horseradish peroxidase‐conjugated secondary antibody (Pierce) for 1 hour at room temperature. The development of the blot was performed using ECL reagent (Amersham, Inc, Piscataway, USA), and the signals were recorded on Kodak Biomax film. The relative levels of each protein were determined by scanning the film and analysing the resulting images.

For immunoprecipitation, the lysate was hatched with the antibody overnight (4°C). Protein G‐sepharose beads (Millipore) were used for incubation for 5 hours (4°C) and then washed with lysis buffer. Protein immunoprecipitated was resolved by SDS‐PAGE and probed using primary antibodies and secondary antibodies.

### 2.12 Double immunofluorescence staining and double Staining of TUNEL and NeuN

Brain slices or cell coverslips were fixed with 4% paraformaldehyde for 30 minutes, and the cell membrane was permeabilized with 0.1% Triton X‐100 for 10 minutes at room temperature. 5% normal goat serum in PBS was used for blockade for 1 hour at room temperature. Then primary antibodies, TNF‐α, Caspase3, pBad, glial fibrillary acidic protein (GFAP), MAP2 (Abcam, Cambridge, MA, USA), were used for overnight incubation at 4°C. Alexa Fluor 488 (green)/Alexa Fluor 594 (red) conjugated secondary antibody (Abcam) was performed to bind to the primary antibodies for 30 min at room temperature.

According to the manufacturer's protocol for commercial kits (Roche, Basel, Switzerland), terminal‐deoxynucleotidyl transferase mediated nick end labeling (TUNEL) staining was performed with NeuN antibodies (Abcam) and 496‐diamidino‐2phenylindole (Beyotime, Shanghai, China).

### 2.13 Statistical analysis

The expression of data was shown as mean ± SD. Analysis of data comparison was performed using one‐way ANOVA. Under the condition of using the envelope test to test the normal distribution, if the variances were determined equal by *F* test, all the data were tested with normal distribution and mean square error. In the face of significant differences detected by the ANOVA model, Dunnett's post hoc multiple comparison test was applied. P value is adjusted by Bonferroni correction. Significant levels were determined when *P* < 0.05 or *P* < 0.01. SPSS 18.0 was used for all analyses (PASW Statistics 18.0).

## 3 RESULTS

### 3.1 NaHS improved DA‐induced cognitive impairment in MHE rats

**FIGURE 1:**
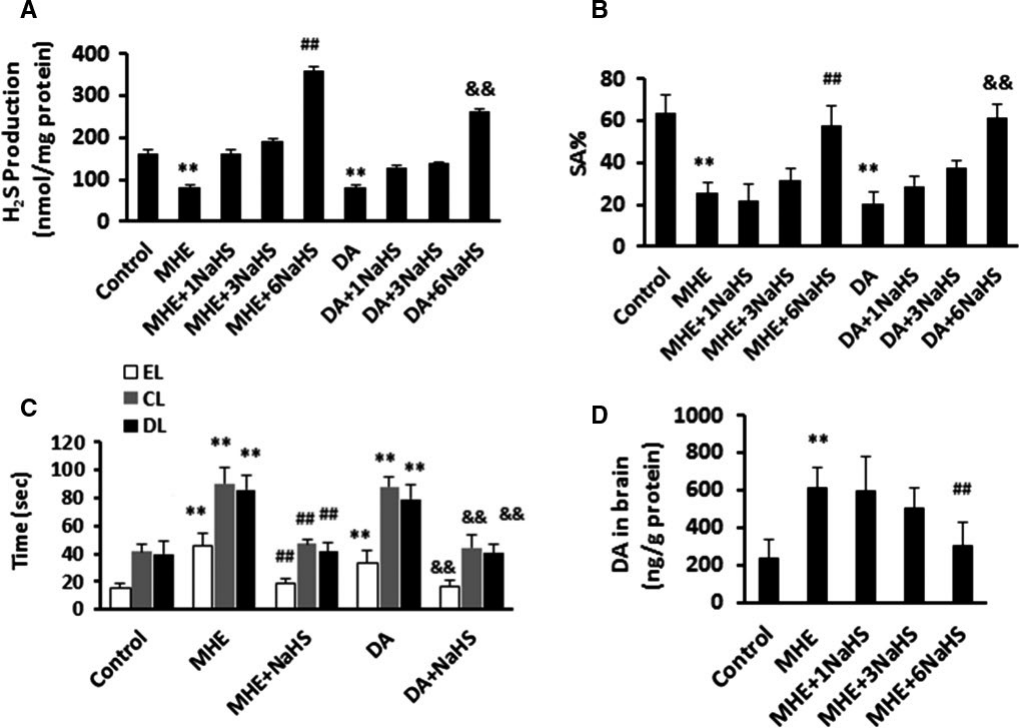
NaHS improved impairment of learning and memory in minimal hepatic encephalopathy (MHE) rats. A, Hydrogen sulphide (H_2_S) detection of homogenates of brain from MHE rats or dopamine (DA)‐treated rats administrated with various concentrations of NaHS (1, 3 or 6 mg/kg) using fluorescent methods. B, Spontaneous alternation percentage (SA%) in Y maze of MHE rats or DA‐treated rats administrated with various concentrations of NaHS (1, 3 or 6 mg/kg). C, Results of WFT (EL, entry latency; CL, contacting latency; DL, drinking latency) of MHE rats or DA‐treated rats administrated with 6 mg/kg NaHS. D, High Performance Liquid Chromatography (HPLC) assay for DA concentrations of homogenates of brain from MHE rats administrated with various concentrations of NaHS (1, 3 or 6 mg/kg). Data are shown as mean ± SD. *P < 0.05, **P < 0.01 vs control rats; ^#^ *P* < 0.05, ^##^ *P* < 0.01 vs MHE rats; ^&^ *P* < 0.05, ^&&^ *P* < 0.01 vs DA‐treated rats

Our goal here is to investigate the potential and beneficial effects of NaHS on DA‐triggered astrocytic TNF‐α release and neuronal toxicity in vitro and in vivo. First, we assessed H_2_S content in MHE rats and DA‐treated rats after NaHS administration. We observed that low dose NaHS (1 or 3 mg/kg) treatment resulted in increased trend of H_2_S production compared to MHE or DA‐treated group; however, H_2_S production was significantly increased in both of MHE‐ and DA‐treated groups after high dose of NaHS treatments (Figure 1A).

Then, we tested whether NaHS improved memory loss in MHE rats and DA‐treated rats. In YM, administration of NaHS increased SA% in MHE rats and DA‐treated rats in a dose‐dependent manner (Figure 1B). In WFT, NaHS treatment (6 mg/kg) induced significant decreases in EL, CL and DL in MHE rats and DA‐treated rats (Figure 1C). These data indicate that NaHS rescues DA‐triggered memory decline in MHE. We also confirmed that DA levels in the brains were obviously increased in MHE rats, whereas NaHS administration significantly reduced DA levels (Figure 1D). Of note, our study presents that H_2_S is sufficient to ameliorate DA‐driven memory impairment by inhibiting inflammation and neuronal apoptosis.

### 3.2 NaHS decreased DA‐mediated astrocytic TNF‐α release and p65 acetylation in vitro

**FIGURE 2:**
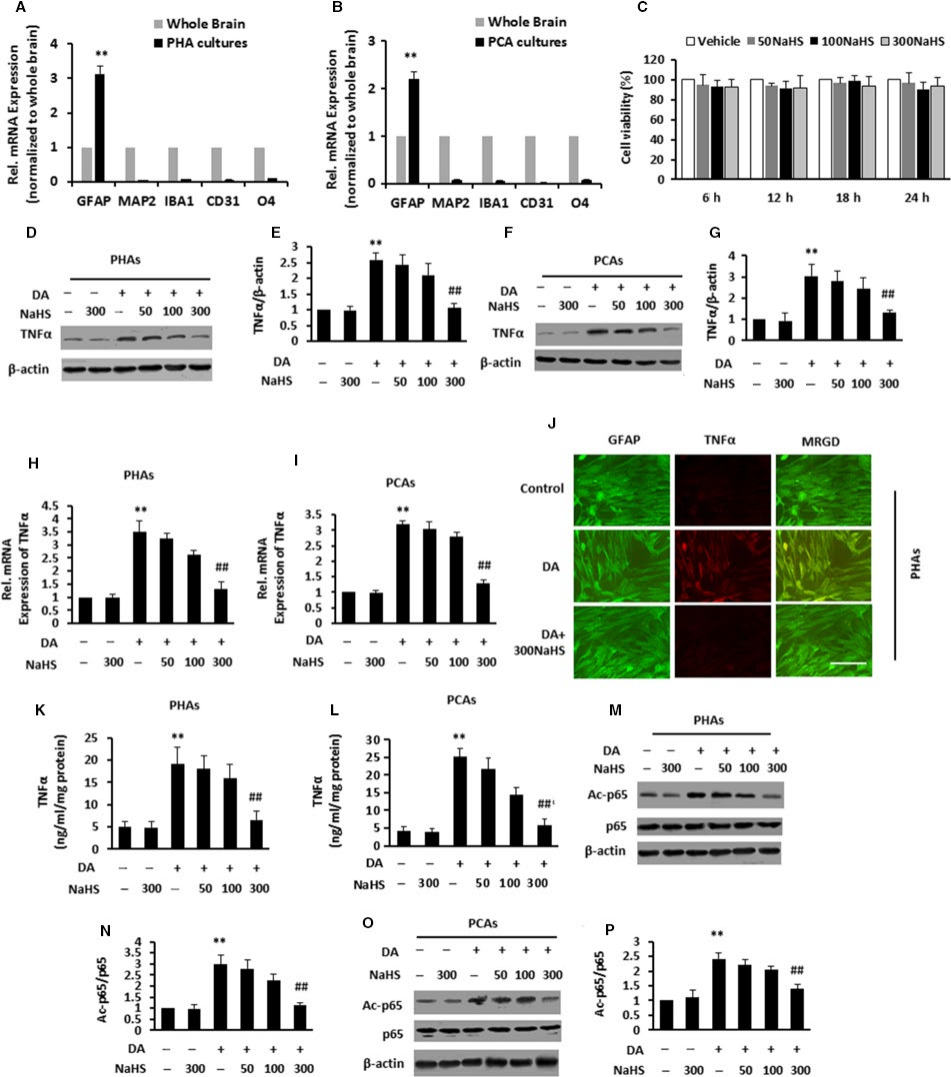
NaHS decreased dopamine (DA)‐mediated astrocytic tumour necrosis factor‐α (TNF‐α) release and acetylation of p65 in vitro. A and B, Cell specific genes were measured by Quantitative Real‐time polymerase chain reaction (qPCR) in PHAs (A) or PCAs (B) cultures compared to expression levels in whole brain homogenates. C, MTT assay for PHAs stimulated with various concentrations of NaHS (50, 100 or 300 μmol/L). D‐G, Immunoblot analysis and subsequent densitometry analysis of lysates from PHAs (d, e) or PCAs (f, g) stimulated with various concentrations of NaHS (50, 100 or 300 μmol/L) after pre‐incubation with DA using anti‐TNF‐α and anti‐β‐actin antibodies. H and I, PHAs (H) or PCAs (I) stimulated with various concentrations of NaHS (50, 100 or 300 μmol/L) after pre‐incubation with DA were analysed for TNF‐α mRNAs by qPCR. J, Double immunofluorescence staining of PHAs stimulated with various concentrations of 300 μmol/L NaHS after pre‐incubation with DA using antibodies against TNF‐α (red), GFAP (green). K and L, ELISA assay for TNF‐α levels of supernatant from PHAs (K) or PCAs (L) stimulated with various concentrations of NaHS (50, 100 or 300 μmol/L) after pre‐incubation with DA. M‐P, Immunoblot and subsequent densitometry analysis of lysates from PHAs (M, N) or PCAs (O, P) stimulated with various concentrations of NaHS (50, 100 or 300 μmol/L) after pre‐incubation with DA using anti‐Ac‐p65/p65 and anti‐β‐actin antibodies. Data are shown as mean ± SD. *P < 0.05, **P < 0.01 vs vehicle group; ^#^ *P* < 0.05, ^##^ *P* < 0.01 vs DA‐treated group. Scale bar, 25 μm. MRGD, merged image

We first examined our cell culture by RT‐PCR using targeting specific cell markers including astrocytes (GFAP), endothelial cells (CD31), microglia (IBA1), neurons (MAP2) and oligodendrocytes (O_4_). We found that PHAs (Figure 2A) or PCAs (Figure 2B) had higher expressions of GFAP and lower expressions of other cell type specific genes compared with whole brain. Altogether, these data displayed successfully enriched astrocyte cultures. Because the potential toxicity of NaHS for astrocytes has never been evaluated, we then examined whether NaHS affected cell death by the MTT assay. 0‐300 μmol/L NaHS did not exhibit any toxic effects on the PHAs viability (Figure 2C).

Dopamine has previously been reported to induce TNF‐α release from astrocytes.^19^ Thus, we tested the impact of NaHS on DA‐induced TNF‐α release from astrocytes in vitro. By IB analysis, NaHS treatment markedly reduced TNF‐α release in a dose‐dependent manner from PHAs (Figure 2D,E) or PCAs (Figure 2F,G) in response to DA. Based on qPCR, NaHS treatment reduced the DA‐induced increase of TNF‐α mRNA in a dose‐dependent fashion in PHAs (Figure 2H) and PCAs (Figure 2I). As indicated by the IF analysis, we confirmed that TNF‐α expression was reduced by the addition of NaHS to DA‐treated PHAs (Figure 2J). NaHS treatment also dose‐dependently decreased the release of TNF‐α from DA‐treated PHAs (Figure 2K) or DA‐treated PCAs (Figure 2L), as determined by ELISA.

The transcription factor nuclear factor kappa‐B (NF‐kB) played a key role in regulating inflammatory activity by the acetylation of NF‐kB subunit p65/RelA.^22^ We investigated whether NaHS decreased TNF‐α production by inhibiting p65 acetylation. IB analysis showed that DA significantly increased p65 acetylation in PHAs, while high dose of NaHS treatment obviously reversed the effect of DA, compared with unstimulated cells (Figure 2M,N). The increased p65 acetylation induced by DA treatment in PCAs was blocked by the addition of high dose of NaHS (Figure 2O,P). Our study showed that NaHS treatment could markedly inhibit DA‐induced pro‐inflammatory cytokine release.

### 3.3 NaHS suppressed TNF‐α‐induced neuronal apoptosis in vitro

**FIGURE 3:**
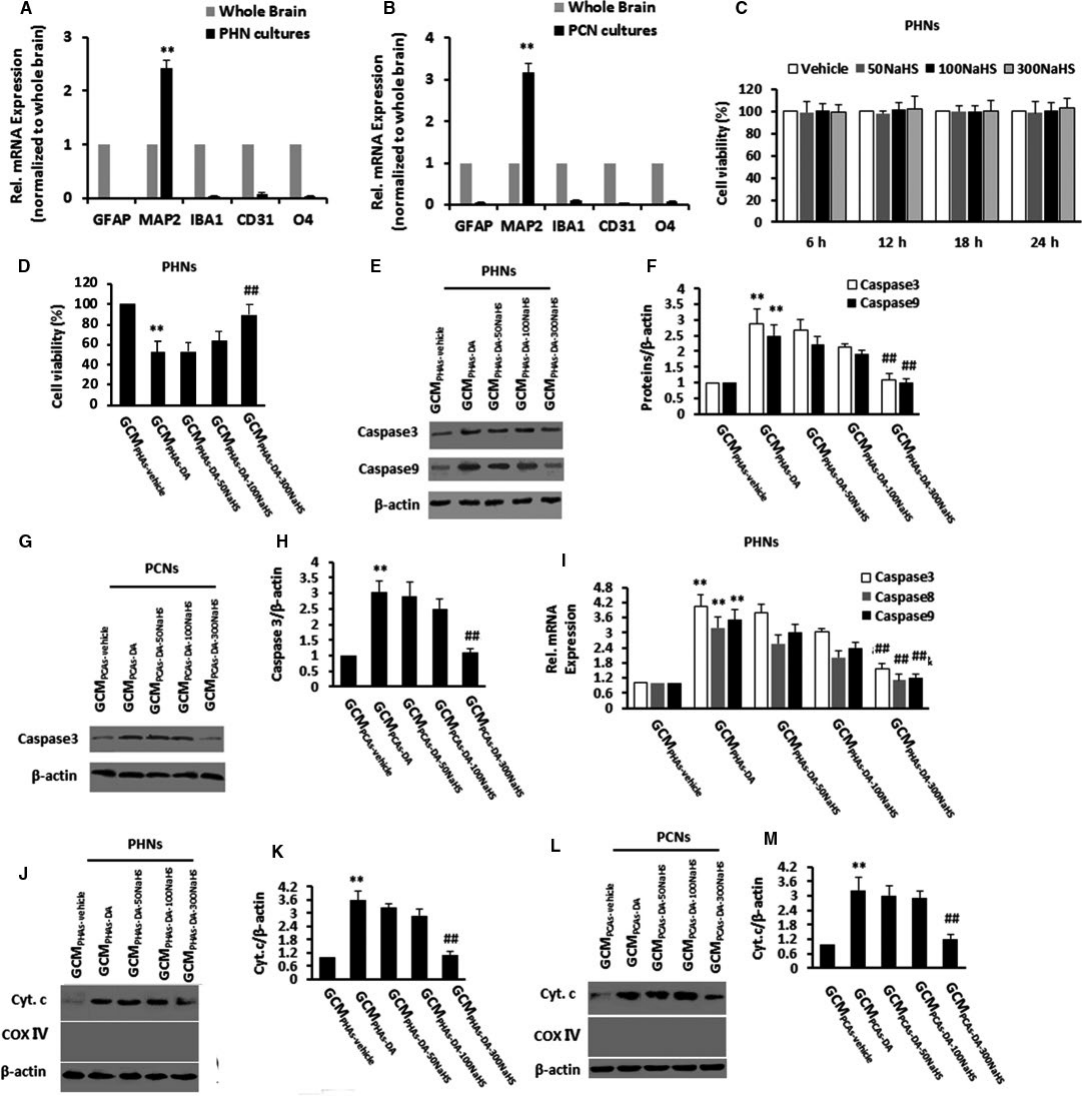
NaHS decreased astrocytic tumour necrosis factor‐α release to inhibit apoptosis in primary cultured neurons. A and B, Cell specific genes were measured by qPCR in PHNs (A) or PCNs (B) cultures compared to expression levels in whole brain homogenates. C, MTT assay for PHNs stimulated with various concentrations of NaHS (50, 100 or 300 μmol/L). D, MTT assay for PHNs stimulated with conditioned glial medium (GCM) from PHAs with vehicle treatment (GCM_PHAs‐vehicle_), GCM from PHAs with 10 μmol/L DA treatment (GCM_PHAs‐DA_), GCM from PHAs with 10 μmol/L DA + 50 μmol/L NaHS treatment (GCM_PHAs‐DA‐50NaHS_), GCM from PHAs with 10 μmol/L DA + 100 μmol/L NaHS treatment (GCM_PHAs‐DA‐100NaHS_), GCM from PHAs with 10 μmol/L DA + 300 μmol/L NaHS treatment (GCM_PHAs‐DA‐300NaHS_). E and F, Immunoblot and subsequent densitometry analysis of PHNs stimulated with GCM_PHAs‐vehicle_, GCM_PHAs‐DA_, GCM_PHAs‐DA‐50NaHS_, GCM_PHAs‐DA‐100NaHS_, GCM_PHAs‐DA‐300NaHS_ using anti‐Caspase3/9 and anti‐β‐actin antibodies. G and H, Immunoblot and subsequent densitometry analysis of PCNs stimulated with GCM_PCAs‐vehicle_, GCM_PCAs‐DA_, GCM_PCAs‐DA‐50NaHS_, GCM_PCAs‐DA‐100NaHS_, GCM_PCAs‐DA‐300NaHS_ using anti‐Caspase3 and anti‐β‐actin antibodies. I, PHNs stimulated with GCM_PHAs‐vehicle_, GCM_PHAs‐DA_, GCM_PHAs‐DA‐50NaHS_, GCM_PHAs‐DA‐100NaHS_, GCM_PHAs‐DA‐300NaHS_ were analysed for Caspase3/8/9 mRNAs by qPCR. J and K, Immunoblot and subsequent densitometry analysis of PHNs stimulated with GCM_PHAs‐vehicle_, GCM_PHAs‐DA_, GCM_PHAs‐DA‐50NaHS_, GCM_PHAs‐DA‐100NaHS_, GCM_PHAs‐DA‐300NaHS_ using anti‐Cyt.c and anti‐Cox Ⅳ/β‐actin antibodies. L and M, Immunoblot and subsequent densitometry analysis of PCNs stimulated with GCM_PCAs‐vehicle_, GCM_PCAs‐DA_, GCM_PCAs‐DA‐50NaHS_, GCM_PCAs‐DA‐100NaHS_, GCM_PCAs‐DA‐300NaHS_ using anti‐Cyt.c/Cox Ⅳ and anti‐β‐actin antibodies. Data are shown as mean ± SD. *P < 0.05, **P < 0.01 vs whole brain/GCM_PHAs‐vehicle_/GCM_PCAs‐vehicle_‐treated group; ^#^ *P* < 0.05, ^##^ *P* < 0.01 vs GCM_PHAs‐DA_/GCM_PCAs‐DA_‐treated group

It has been reported that neurodegeneration in MHE is triggered by DA depending on astrocytic TNF‐α mediation.^19^ We first tested our cell culture through RT‐PCR using targeting specific cell markers. We found that PHNs (Figure 3A) or PCNs (Figure 3B) had higher expressions of MAP2 and lower expressions of other cell type specific genes compared to the whole brain. Altogether, these data showed successfully enriched neuron cultures. We then examined the potential toxicity of NaHS on cell viability of neurons by the MTT assay, but 0‐300 μmol/L NaHS had no effect on the PHNs death (Figure 3C).

Tumour necrosis factor‐α, not DA, was reported to directly induce neuronal apoptosis.^19^ Thus, we tested whether NaHS had an effect on TNF‐α‐stimulated neuronal apoptosis by TUNEL staining and immunoblotting using caspase 3/9 and Cyt. c. Based on MTT assay, PHNs exhibited significant cell death in response to GCM_PHAs‐DA_ treatment compared with PHNs in response to GCM_PHAs‐vehicle._ GCM_PHAs‐DA‐300NaHS_ treatment markedly reversed GCM_PHAs‐DA_ treatment‐induced PHNs death, showing increased cell viability (Figure 3D). PCNs showed elevated Caspase3/9 levels in response to GCM_PCAs‐DA_ treatment compared to the GCM_PCAs‐vehicle_‐treated group_._ GCM_PCAs‐DA‐300NaHS_ treatment reversed the levels of Caspase3/9 in PCNs compared with GCM_PCAs‐DA_ treatment and GCM_PCAs‐DA‐50NaHS_/GCM_PCAs‐DA‐100NaHS_ treatment induced a modest Caspase3/9 level change (Figure 3E,F). PHNs exhibited increased Caspase3 levels in response to GCM_PHAs‐DA_ treatment compared to PHNs in response to GCM_PHAs‐vehicle_, whereas GCM_PHAs‐DA‐300NaHS_ treatment reversed the levels of Caspase3 in PHNs compared with GCM_PHAs‐DA_ treatment in a dose‐dependent fashion (Figure 3G,H). By qPCR, GCM_PCAs‐DA_ treatment increased Caspase3/8/9 mRNA in PHAs, while GCM_PHAs‐DA‐NaHS_ treatment reversed the effect of GCM_PHAs‐DA_ in a dose‐dependent fashion (Figure 3I). Of note, GCM_PHAs‐DA_ treatment triggered mitochondrial Cyt. c release to cytoplasm in PHNs, whereas GCM_PHAs‐DA‐300 NaHS_ treatment reduced Cyt. c release (Figure 3J,K). We also observed increased cytoplasmic Cyt. c in PCNs with GCM_PCAs‐DA_ treatment, which was dramatically reversed by the GCM_PCAs‐DA‐300 NaHS_ treatment (Figure 3L,M). These data confirmed that NaHS ameliorated apoptosis by reduction of TNF‐α level.

**FIGURE 4:**
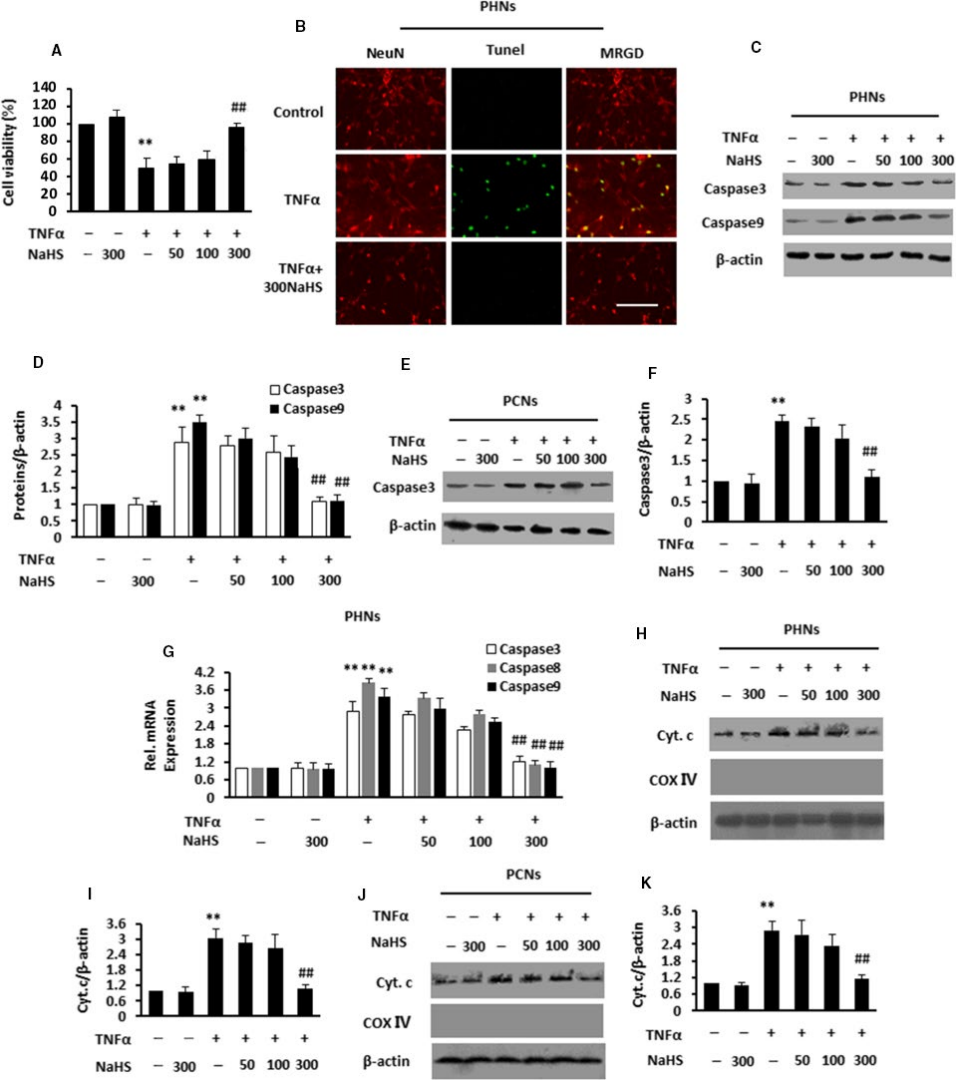
NaHS directly suppressed tumour necrosis factor‐α (TNF‐α)‐induced neuronal apoptosis in primary cultured neurons. A, MTT assay for PHNs stimulated with various concentrations of NaHS (50, 100, 300 μmol/L) after pre‐incubation with TNF‐α. B, Visualization of apoptotic cell nuclei (green), neurons (red) in PHNs stimulated with various concentrations of 300 μmol/L NaHS after pre‐incubation with TNF‐α by TUNEL staining and anti‐NeuN staining. C and D, Immunoblot and subsequent densitometry analysis of PHNs stimulated with various concentrations of NaHS (50, 100, 300 μmol/L) after pre‐incubation with TNF‐α using anti‐Caspase3/9 and anti‐β‐actin antibodies. E and F Immunoblot and subsequent densitometry analysis of PCNs stimulated with various concentrations of NaHS (50, 100, 300 μmol/L) after pre‐incubation with TNF‐α using anti‐Caspase3 and anti‐β‐actin antibodies. G, PHNs stimulated with various concentrations of NaHS (50, 100, 300 μmol/L) after pre‐incubation with TNF‐α were analysed for Caspase3/8/9 mRNAs by qPCR. H‐K, Immunoblot and subsequent densitometry analysis of PHNs (H, I) or PCNs (J, K) stimulated with various concentrations of NaHS (50, 100, 300 μmol/L) after pre‐incubation with TNF‐α using anti‐Cyt.c/Cox Ⅳ and anti‐β‐actin antibodies. Data are shown as mean ± SD. *P < .05, **P < 0.01 vs vehicle‐treated group; ^#^ *P* < 0.05, ^##^ *P* < 0.01 vs TNF‐α‐treated group. Scale bar, 25 μm. MRGD, merged image

We next investigated whether NaHS directly attenuated TNF‐α‐induced neuronal apoptosis. MTT assay showed that TNF‐α significantly induced cell death in PHNs and NaHS abrogated the effect of TNF‐α (Figure 4A). PCNs showed a significant elevated signal by TUNEL staining in response to TNF‐α, which was abolished by NaHS (Figure 4B). As determined by the IB analysis in Figure 4C,D, we confirmed an up‐regulated Caspase3/9 expression in PHNs exposed to TNF‐α and NaHS addition decreased the Caspase3/9 expression in a dose‐dependent manner. Caspase3 expression was also elevated in PCNs exposed to TNF‐α, which was blocked by the addition of 300 μmol/L NaHS (Figure 4E,F). Based on qPCR, TNF‐α treatment increased Caspase3/8/9 mRNA in PHAs or PCAs, and NaHS treatment reversed the effect of TNF‐α in a dose‐dependent fashion (Figure 4G). Tumour necrosis factor‐α stimulated mitochondria to secrete cellular Cyt.c to the cytoplasm, which was dramatically reversed by the 300 μmol/L NaHS treatment in PHNs (Figure 4H,I) or PCNs (Figure 4J,K). We confirmed that the treatment with GCM_PHAs‐DA‐300NaHS_ or GCM_PCAs‐DA‐300NaHS_, in which TNF‐α content was significantly decreased by NaHS, inhibited apoptosis of neurons.

### 3.4 NaHS blocked TNF‐α‐induced dephosphorylation of Bad via S6K1 in neurons

**FIGURE 5:**
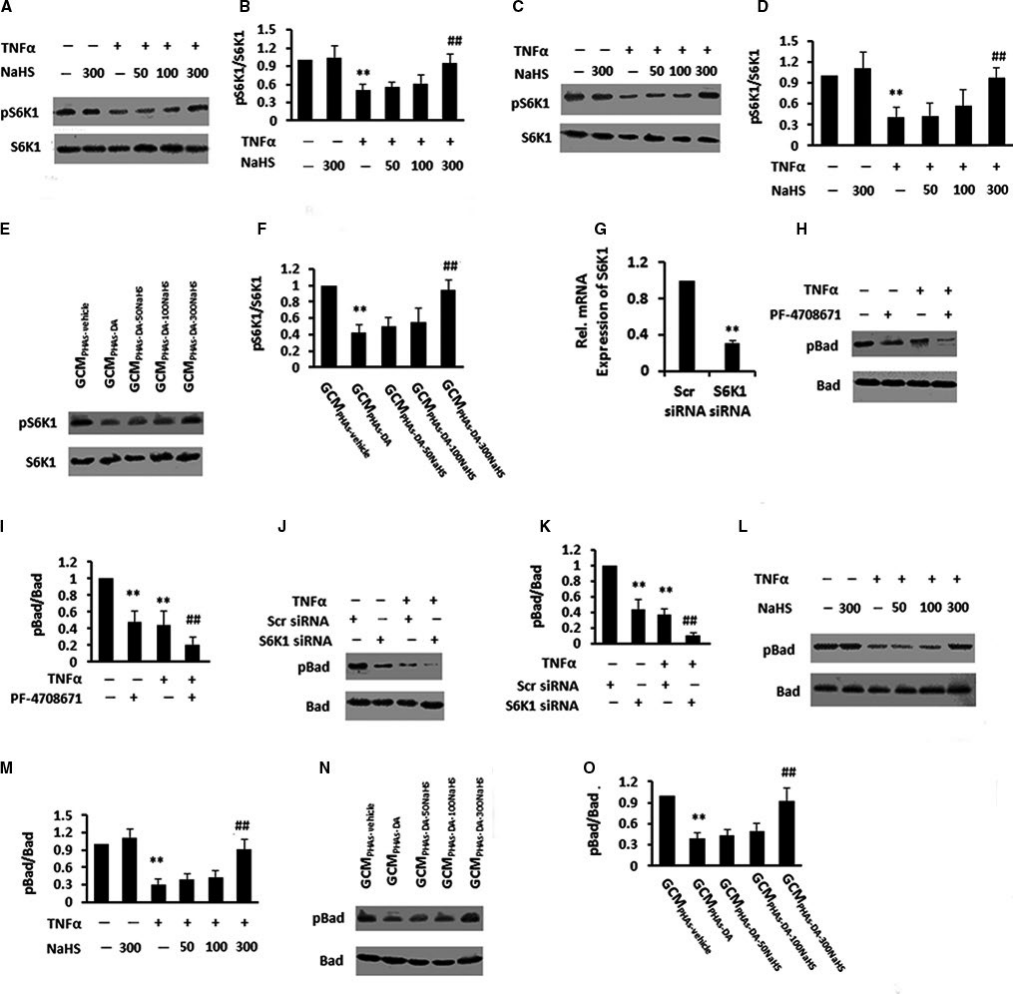
NaHS reduced tumour necrosis factor‐α (TNF‐α)‐induced dephosphorylation of Bad via S6K1 in primary cultured neurons. A‐D, Immunoblot and subsequent densitometry analysis of lysates from PHNs (A, B) and PCNs (C, D) stimulated with various concentrations of NaHS (50, 100, 300 μmol/L) after pre‐incubation with TNF‐α using anti‐pS6K1/S6K1 antibodies. E and F, Immunoblot and subsequent densitometry analysis of PHNs stimulated with GCM_PHAs‐vehicle_, GCM_PHAs‐DA_, GCM_PHAs‐DA‐50NaHS_, GCM_PHAs‐DA‐100NaHS_, GCM_PHAs‐DA‐300NaHS_ using anti‐pS6K1/S6K1 antibodies. Data are shown as mean ± SD. *P < 0.05, **P < 0.01 vs vehicle/GCM_PHAs‐vehicle_‐treated group; ^#^ *P* < 0.05, ^##^ *P* < 0.01 vs TNF‐α/GCM_PHAs‐DA_‐treated group. G, PHNs with S6K1 siRNA transfection were analysed for S6K1 mRNAs by qPCR. H and I, Immunoblot and subsequent densitometry analysis of lysates from PHNs stimulated with TNF‐α after pre‐incubation with PF‐4708671 using antibodies against pBad/Bad. J and K, Immunoblot and subsequent densitometry analysis of lysates from PHNs stimulated with DA after S6K1 siRNA transfection using antibodies against pBad/Bad. L and M, Immunoblot and subsequent densitometry analysis of lysates from PHNs stimulated with various concentrations of NaHS (50, 100, 300 μmol/L) after pre‐incubation with TNF‐α using anti‐pBad/Bad antibodies. N and O, Immunoblot analysis Immunoblot analysis of lysates from PHNs stimulated with GCM_PHAs‐vehicle_, GCM_PHAs‐DA_, GCM_PHAs‐DA‐50NaHS_, GCM_PHAs‐DA‐100NaHS_, GCM_PHAs‐DA‐300NaHS_ using anti‐pBad/Bad antibodies. Data are shown as mean ± SD. *P < 0.05, **P < 0.01 vs vehicle/Scr siRNA/GCM_PHAs‐vehicle_‐treated group; ^#^ *P* < 0.05, ^##^ *P* < 0.01 vs TNF‐α/TNF‐α + Scr siRNA/GCM_PHAs‐DA_‐treated group. GCM, conditioned glial medium

S6K1 might have been implicated in regulating cell death and survival.^23^ Here, we investigated whether NaHS affected S6K1's phosphorylation. However, as determined by IB analysis, we found decreased phosphorylation of S6K1 in PHNs with TNF‐α treatment, while 300 μmol/L NaHS induced the reversal of the effect of TNF‐α (Figure 5A,B). We further found that the addition of NaHS also increased pS6K1 levels in PCNs in response to TNF‐α (Figure 5C,D), indicating direct stimulation of the phosphorylation of S6K1 by NaHS in neurons in response to TNF‐α. Otherwise, GCM_PHAs‐DA_ treatment reduced the phosphorylation of S6K1 as compared to PHNs in response to GCM_PHAs‐vehicle,_ and GCM_PHAs‐DA‐300NaHS_ treatment markedly increased the levels of pS6K1 in PHNs compared with GCM_PHAs‐DA_ treatment (Figure 5E,F), indicating activation of S6K1 pathway by NaHS through reduction of TNF‐α release from astrocytes.

Bad, as a pro‐apoptotic member of BCL‐2 family, once Phosphorylated, was involved in the maintenance of cell survival.^24^, ^25^ We assessed whether NaHS elicited phosphorylation of Bad through phosphorylation cascade of S6K1 using S6K1 inhibition and siRNA‐induced silencing. We first tested the efficiency of S6K1 siRNA transfection into MHE astrocytes. As determined by qPCR, PHNs showed weak S6K1 after S6K1 siRNA transfection (Figure 5G), as compared to control siRNA transfection, indicating efficient transfection. Figure 5H,I showed decreases in the phosphorylation of Bad after TNF‐α treatment, whereas the effect of TNF‐α was enhanced by S6K1 inhibitor PF‐4708671 in PHNs. Additionally, knockdown of S6K1 also induced the enhancement of TNF‐α‐induced decrease in pBad level in PHNs (Figure 5J,K).

We then asked whether NaHS impacted on the phosphorylated Bad. The addition of NaHS significantly blocked TNF‐α‐mediated decrease in pBad levels in PHNs (Figure 5L,M), indicating a direct stimulation of the phosphorylation of Bad by NaHS in neurons in response to TNF‐α. Then, we examined the role of GCM_PHAs‐DA‐NaHS_ on the phosphorylation of Bad. PHNs treated with GCM_PHAs‐DA_ showed a reduction of pBad levels compared with GCM_PHAs‐vehicle_‐treated group; however, GCM_PHAs‐DA‐300NaHS_ treatment markedly increased the levels of pBad in PCNs compared with GCM_PCAs‐DA_ treatment, indicating the involvement of reduced TNF‐α level in the improvement of apoptosis (Figure 5N,O).

NaHS induced the phosphorylation of Bad, and the interaction between Bclxl and pro‐apoptotic protein Bak, resulting in the inhibition of mitochondria‐mediated apoptosis.

### 3.5 NaHS altered TNF‐α‐induced Bad‐Bclxl‐Bak interactions in neurons

**FIGURE 6:**
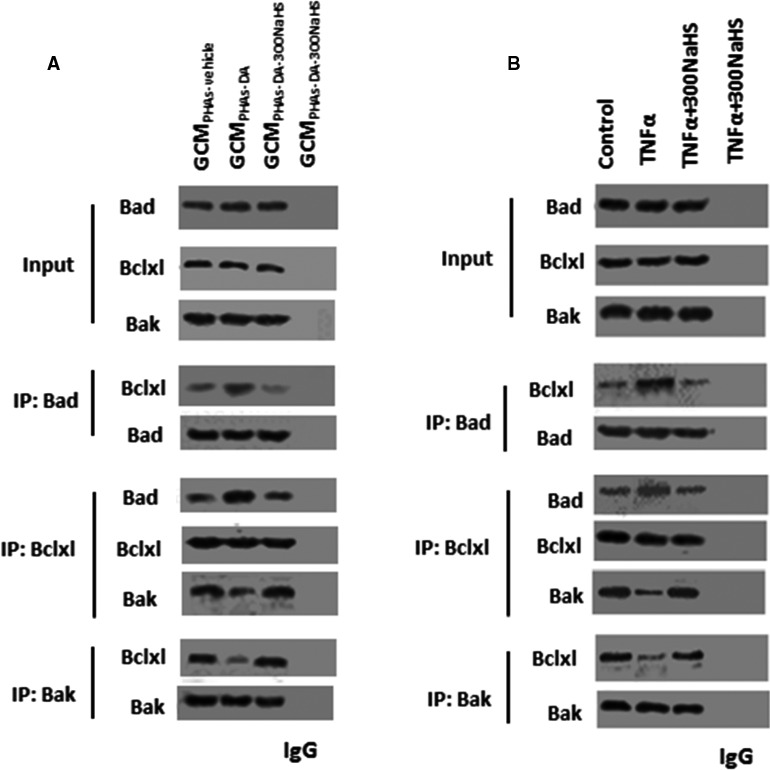
The effect of NaHS on tumour necrosis factor‐α (TNF‐α) for the alterations of Bad‐Bclxl‐Bak interaction in primary cultured neurons. A, Immunoprecipitation (IP) of lysates from PHNs stimulated with GCM_PHAs‐vehicle_, GCM_PHAs‐DA_, GCM_PHAs‐DA‐300NaHS_ with control IgG, anti‐Bad/Bclxl/Bak antibodies. Complexes were immunoblotted with anti‐Bad/Bclxl/Bak antibodies. B, IP of lysates from PHNs stimulated with various concentrations of 300 μmol/L NaHS after pre‐incubation with TNF‐α with control immune globulin G (IgG) and anti‐Bad/Bclxl/Bak antibodies. Complexes were immunoblotted with anti‐Bad/Bclxl/Bak antibodies. GCM, conditioned glial medium

The phosphorylation of Bad elicited the disruption of the binding of Bad to Bclxl and enhancement of the binding of Bak to Bclxl, leading to cell survival.^24^, ^25^, ^26^, ^27^ Thus, we assessed whether GCM_PHAs‐DA‐300NaHS_ played a role in the association of Bak and Bclxl or Bclxl and Bad by co‐immunoprecipitation. As determined in Figure 6A, GCM_PHAs‐DA_ treatment induced an increase in Bclxl level that co‐immunoprecipitated with Bad, which was blocked by GCM_PHAs‐DA‐300NaHS_ treatment. GCM_PHAs‐DA_ treatment reduced the amount of Bak and increased the amount of Bad that co‐immunoprecipitated with Bclxl, which was also abrogated by GCM_PHAs‐DA‐300NaHS_ treatment. GCM_PHAs‐DA_ treatment also reduced the level of Bclxl that co‐immunoprecipitated with Bak, which was also diminished by GCM_PHAs‐DA‐300NaHS_ treatment. The results suggested that NaHS stimulated the Bak‐Bclxl interaction and disrupted Bclxl‐Bad interaction in neurons through reduction of TNF‐α release from astrocytes.

Then, we addressed the impact of NaHS on TNF‐α‐mediated Bak‐Bclxl or Bclxl‐Bad interaction. As shown in Figure 6B, TNF‐α increased Bclxl level that co‐immunoprecipitated with Bad, which was blocked by NaHS. TNF‐α also triggered decreased levels of Bax and increased level of Bad that co‐immunoprecipitated with Bclxl, which was also diminished by NaHS. The level of Bclxl that co‐immunoprecipitated with Bak was significantly decreased by TNF‐α, which was also abated by NaHS.

Taken together, we suggested that NaHS protected against apoptosis, possibly through the phosphorylation of Bad, which induced dissociation of Bad from mitochondrial Bclxl followed by the interaction between Bak and mitochondrial Bclxl, and led to anti‐apoptosis.

### 3.6 NaHS attenuated DA‐stimulated inflammatory response and neurodegeneration in MHE rats

**FIGURE 7:**
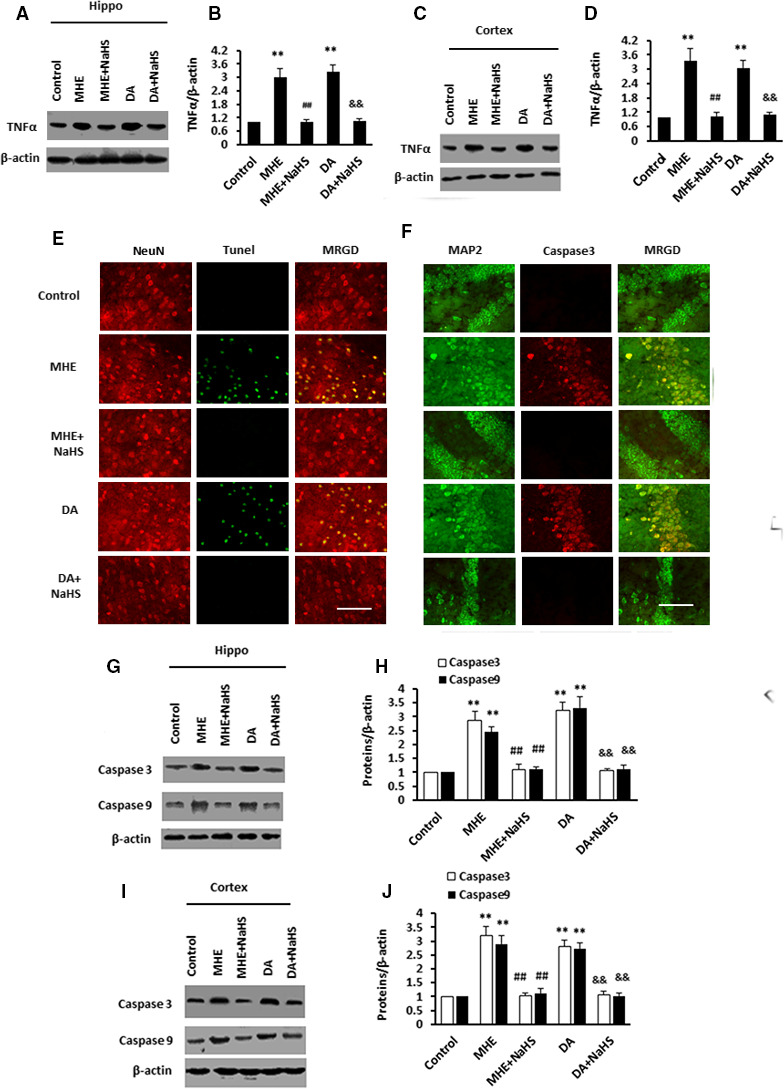
NaHS lowered dopamine (DA)‐induced astrocytic tumour necrosis factor‐α (TNF‐α) production and neurodegeneration in minimal hepatic encephalopathy (MHE) rats. A‐D, Immunoblot and subsequent densitometry analysis of hippocampal (A, B) or cortical (C, D) lysates from MHE rats or DA‐treated rats administrated with NaHS using anti‐TNF‐α/β‐actin antibodies. E, Visualization of apoptotic cell nuclei (green), neurons (red) in the cortex sections from MHE rats or DA‐treated rats administrated with NaHS by TUNEL staining and anti‐NeuN staining. F, Double immunofluorescence staining of the hippocampus sections from MHE rats or DA‐treated rats administrated with NaHS using antibodies against Caspase3 (red) and MAP2 (green). G‐J, Immunoblot and subsequent densitometry analysis of hippocampal (G, H) or cortical (I, J) lysates from MHE rats or DA‐treated rats administrated with NaHS using anti‐Caspase3/9 and β‐actin antibodies. Data are shown as mean ± SD. *P < 0.05, **P < 0.01 vs control rats; ^#^ *P* < 0.05, ^##^ *P* < 0.01 vs MHE rats; ^&^ *P* < 0.05, ^&&^ *P* < 0.01 vs DA‐treated rats. Scale bar, 25 μm. Hippo, hippocampus; MRGD, merged image

We then assessed the impact of NaHS on the inflammatory signalling pathway in vivo. A significant increase in TNF‐α expression was observed in the hippocampus of MHE and DA‐treated rats, while NaHS treatment improved the expression of TNF‐α (Figure 7A,B). The expression of TNF‐α was also significantly increased in the cortex in MHE and DA‐treated rats, while NaHS treatment reversed the down‐regulation of TNF‐α (Figure 7C,D). These data suggested that NaHS ameliorated DA‐triggered inflammatory response in astrocytes in MHE rats.

We further tested the role of NaHS on the apoptotic caspase signalling pathway in vivo. The hippocampus of MHE‐ and DA‐treated rats showed an increase in TUNEL staining in neurons (Figure 7E), which was reversed by administration of NaHS. Based on immunofluorescence staining, Caspase3 was strongly expressed in neurons in the hippocampus of MHE‐ and DA‐treated rats but was weakly expressed after the administration of NaHS (Figure 7F). Immunoblotting confirmed that Caspase3/9 expressions were increased in the hippocampus (Figure 7G,H) of MHE and DA‐treated rats, while Caspase3/9 levels were markedly decreased by NaHS. The cortexes (Figure 7I,J) exhibited elevations of Caspase3/9 in MHE‐ and DA‐treated rats_,_ and NaHS administration markedly reversed the levels of Caspase3/9, indicating an involvement of Caspase3/9 in reducing TNF‐α level to reduce apoptosis. It is documented that NaHS suppressed DA‐induced TNF‐α production to inhibit neuronal apoptosis in astrocytes and in vivo.

## 4 DISCUSSION

Astrocytes are now emerging as pivotal regulators of potent pro‐inflammatory responses in the central nervous system.^28^, ^29^ Several studies reported that DA overload‐induced astrocytic TNF‐α production promoted neuronal apoptosis, leading to the impairment of learning and working memory in the process of MHE pathologies.^18^, ^19^, ^21^, ^30^ Our goal here is to investigate the potential and beneficial effects of NaHS on DA‐triggered astrocytic TNF‐α release and neuronal toxicity in vitro and in vivo. We now found that the treatment with GCM_PHAs‐DA‐300NaHS_ or GCM_PCAs‐DA‐300NaHS_, in which TNF‐α content was significantly decreased by NaHS, suppresses the apoptosis of neurons. Of note, H_2_S is sufficient to reduce inflammation and neuronal apoptosis to ameliorate DA‐driven memory impairment. We also presented here that NaHS directly inhibited neuronal apoptosis induced by TNF‐α. Our study focused on novel aspects of two anti‐apoptotic ways of H_2_S: (a) reduction of DA‐induced astrocytic TNF‐α release; and (b) direct inhibition of effect of TNF‐α on neurons.

An NF‐κB subunit p65 can either potentiate or diminish NF‐kB signalling by regulating the particular acetylated lysine residues,^22^, ^31^, ^32^, ^33^ which is involved in producing pro‐inflammatory cytokines to modulate the developments of age‐related diseases.^33^, ^34^, ^35^, ^36^ Our data showed that DA promoted acetylation of RelA/p65 and increased pro‐inflammatory cytokine levels in astrocytes. Once p65 is acetylated and activated, NF‐ƙB will persistently be activated for the production of TNF‐α in response to DA exposure, which could be reversed by NaHS treatment.

Bad, a BH3 domain‐only pro‐apoptotic member of the Bcl‐2 family, couples death signals in mitochondria to induce apoptosis.^37^ But the phosphorylation of Bad appears to dissociate with Bclxl or Bcl‐2 to induce survival.^24^, ^25^ Moreover, S6K1 controls cell survival, which phosphorylates and inactivates Bad.^38^, ^39^, ^40^ In our current study, TNF‐α was shown to sufficiently suppress phosphorylation of Bad via dephosphorylation of S6K1 and Bad, which resulted in the association of Bad‐Bclxl and dissociation of Bak‐Bclxl, leading to mitochondria dysfunction and caspase cascade. However, in other circumstances, NaHS induced sustained S6K1 activation and phosphorylated Bad in the neurons following application of TNF‐α. The treatment with GCM_PHAs‐DA‐300NaHS_ or GCM_PCAs‐DA‐300NaHS_, in which TNF‐α content was significantly decreased by NaHS, promoted the phosphorylations of S6K1 and Bad, the dissociation of Bad‐Bclxl and association of Bak‐Bclxl in neurons. Thus, NaHS induced the activation and phosphorylation of S6K1, which triggered phosphorylation of Bad, and dissociation of Bad from mitochondrial Bclxl followed by interaction of Bak with mitochondrial Bclxl, resulting in the inhibition of mitochondria‐mediated apoptosis.

In summary, it is documented that NaHS suppressed DA‐induced TNF‐α production and p65 deacetylation to inhibit neuronal apoptosis in astrocytes and in vivo. Moreover, we also presented here that NaHS directly reduced TNF‐α‐mediated phosphorylation of S6K1/Bad and inactivated the pro‐apoptotic function of Bad induced by TNF‐α in neurons and in vivo. Furthermore, our work provides novel insights into the anti‐inflammatory and anti‐apoptotic dual property of H_2_S for the treatment of MHE.

## CONFLICT OF INTEREST

The authors have declared no conflict of interest.

## AUTHOR CONTRIBUTION

**Weishan Zhuge:** Conceptualization (equal); Writing‐original draft (equal). **Qichuan Zhuge:** Project administration (equal); Writing‐original draft (equal). **Weikan Wang:** Data curation (equal); Writing‐review & editing (equal). **Xiaoai Lu:** Methodology (equal). **Ruiming You:** Methodology (equal); Writing‐review & editing (equal). **Leping Liu:** Data curation (equal); Visualization (equal). **He Yu:** Conceptualization (equal); Visualization (equal). **Jian Wang:** Formal analysis (equal); Resources (equal). **Xuebao Wang:** Investigation (equal); Writing‐review & editing (equal). **Yiru Ye:** Methodology (equal); Visualization (equal). **Saidan Ding:** Conceptualization (equal); Project administration (equal); Supervision (lead).

## Data Availability

The data that support the findings of this study are available from the corresponding author upon reasonable request.
